# Histological Hyperspectral Glioblastoma Dataset (HistologyHSI-GB)

**DOI:** 10.1038/s41597-024-03510-x

**Published:** 2024-06-24

**Authors:** Samuel Ortega, Laura Quintana-Quintana, Raquel Leon, Himar Fabelo, María de la Luz Plaza, Rafael Camacho, Gustavo M. Callico

**Affiliations:** 1 Seafood Industry Department, Norwegian Institute of Food, Fisheries and Aquaculture Research (Nofima), Tromsø, Norway; 2 Institute for Applied Microelectronics, University of Las Palmas de Gran Canaria, Las Palmas de Gran Canaria, Spain; 3 Department of Mathematics and Statistics, UiT The Arctic University of Norway, Tromsø, Norway; 4 Fundación Canaria Instituto de Investigación Sanitaria de Canarias (FIISC), Las Palmas de Gran Canaria, Spain; 5 Research Unit, Hospital Universitario de Gran Canaria Doctor Negrín, Las Palmas de Gran Canaria, Spain; 6 Department of Pathological Anatomy, Hospital Universitario de Gran Canaria Doctor Negrín, Las Palmas de Gran Canaria, Spain

**Keywords:** Optical spectroscopy, Image processing

## Abstract

Hyperspectral (HS) imaging (HSI) technology combines the main features of two existing technologies: imaging and spectroscopy. This allows to analyse simultaneously the morphological and chemical attributes of the objects captured by a HS camera. In recent years, the use of HSI provides valuable insights into the interaction between light and biological tissues, and makes it possible to detect patterns, cells, or biomarkers, thus, being able to identify diseases. This work presents the HistologyHSI-GB dataset, which contains 469 HS images from 13 patients diagnosed with brain tumours, specifically glioblastoma. The slides were stained with haematoxylin and eosin (H&E) and captured using a microscope at 20× power magnification. Skilled histopathologists diagnosed the slides and provided image-level annotations. The dataset was acquired using custom HSI instrumentation, consisting of a microscope equipped with an HS camera covering the spectral range from 400 to 1000 nm.

## Background & Summary

Hyperspectral (HS) imaging (HSI) is a technology able to measure both the spatial and spectral information of objects or substances, combining the features of spectroscopy and digital imaging in a single imaging modality. Because the absorption, reflection, transmission and scattering of light are unique to each material, this technology allows non-invasive identification of materials. The first use of HSI was for the remote sensing exploration of the Earth’s surface in the 80s^1^. In recent years, this technology has been extended to a wide range of applications, such as precision agriculture^2,3^, food quality inspection^4–6^, industrial sorting of materials^7,8^, art conservation^9,10^, or forensic sciences^11,12^. In medicine, recent research has proven HSI technology to be useful for different clinical applications^13,14^, for example, as a surgical guidance tool^15,16^, as a tool for early diagnosis^17–19^, or as a technology able to measure different biochemical parameters that can be useful for medical practitioners^20–23^.

Digital and computational pathology techniques are intended to provide pathologists with a tool for the quantitative analysis of pathological specimens, reducing inter-observer variability among different pathologists and saving the time of manual examination of histological specimens^24,25^. Recently, some researchers have investigated HSI as a suitable technology for computational pathology in various fields, such as digital staining, colour enhancement, standardization of pathological slides or the exploitation of autofluorescence or immunohistochemistry of histological slides^26^. However, the primary use of HSI in computational pathology is currently in diagnostic research for routine clinical practice. In this context, recent applications have been focused on the diagnosis of cholangiocarcinoma^27,28^, head and neck squamous cell carcinoma^29^, membranous nephropathy^30^, breast cancer^31^, or the classification of leukocyes^32,33^, among others.

The workflow in HS computational pathology research usually involves digitizing the histological slides using HSI instrumentation and extracting information from the HS images that could be useful for diagnostic purposes using various image processing methods. Although a wide variety of techniques are used in the literature to this end, it is difficult to compare the different approaches fairly, mainly due to the lack of publicly available datasets^34^.

In this work, we provide a publicly available dataset of HS images of haematoxylin and eosin (H&E) stained histological slides corresponding to brain tumours, specifically Glioblastoma (GB)^35,36^. To the best of our knowledge there are other databases related to gastric cancer but, this is the first publicly available dataset of HS brain histological images^35^. This dataset is composed of 469 HS images from 13 different patients, with image-level annotations for two different classes (non-tumour or tumour) according to the manual examination of the histological samples. The HS images cover the spectral range from 400 to 1000 nm and were taken at 20× magnification. On the one hand, this dataset can be relevant for researchers interested in HS image classification and other HS image processing techniques, such as spectral unmixing or HS data compression. This dataset was acquired by our research group and all HS images were employed to train different classification algorithms for GB detection which were presented in previous research work^36–38^. In this manuscript, we exclusively present the curated version of the dataset, from which artifacts and labelling errors found in previous publications have been eliminated. Besides the proposed classification techniques, a broad range of potential methods could be explored to evaluate the effectiveness of HSI in enhancing the performance relative to the outcomes obtained with RGB (Red-Green-Blue) images. On the other hand, this dataset can be used by researchers in the field of computational pathology and pathology practitioners to envision the possibilities of this technology for routine clinical practice. In this work, we provide a repository with the HS data, its homologous RGB image and, a snapshot of the original slides showing the region of interest for each HS image. We also provide a comprehensive explanation of the microscopic HS system, its quality validation process, and how the dataset is organized.

## Methods

This section provides a detailed explanation of the methodology employed in previous works^36–38^, This includes a description of the methods used for collecting histological samples, an overview of the microscopic HS system, and the process of acquiring and processing the HS data.

### Histological samples description

**Fig. 1:**
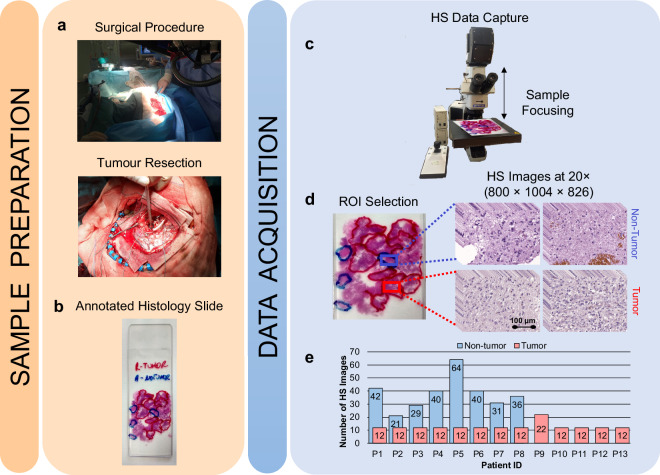
Graphical abstract of the methodology followed. (**a**) Resection procedure. (**b**) Macroscopic annotations of the GB locations. (**c**) HS data capture using a microscopic HS system. (**d**) ROI selection. (**e**) Dataset summary.

The research conducted in this study employs human biopsies extracted during brain tumour resection procedures (Fig. 1a). This research involved participants who were 18 years of age or older, all diagnosed with primary brain tumours and undergoing neurosurgical procedures at the University Hospital of Gran Canaria Doctor Negrín (Las Palmas de Gran Canaria, Spain). Prior to their involvement in the study, each participant provided written informed consent, which explicitly authorized the publication of any images or data obtained during the study. The Research Ethics Committee of the University Hospital of Gran Canaria Doctor Negrin (Comité Ético de Investigación Clínica-Comité de Ética en la Investigación, CEIC/CEI) approved the study protocol and consent procedures (reference 130069). All research procedures were conducted in strict compliance with applicable guidelines and regulations. The pathological slides used in this research were processed and analysed in the Pathological Anatomy Department of the same hospital. After the tumour tissue resection during neurosurgery, the biopsy samples underwent a series of standardized procedures. First, the samples were dehydrated to remove the excess of water, as it is immiscible with most embedding media. The samples were then embedded in paraffin blocks, mounted on microtomes and sliced into 4 *µm* thick slices. Finally, the slices were rehydrated and stained with H&E, a method commonly used in pathology.

The pathologists involved in the study analysed the stained sections using routine examination techniques. Each sample was evaluated and diagnosed as GB (a grade 4 primary brain tumour) according to the 2016 World Health Organization (WHO) classification of tumours of the central nervous system^39^. Macroscopic annotations of the GB locations on the physical pathological slides were made using a red marker pen (Fig. 1b). These annotations served as reference points for further analysis. In addition, non-tumour areas, where no discrete presence of tumour cells was observed, were annotated (blue marker pen) on the histology slides. The pen-marker annotations on the histological slide were deliberately outlined with wide borders to maintain a safety distance between tumour and non-tumour areas. Afterwards, regions of interest (ROIs) were selected from these pathologist-annotated areas for further study. These ROIs were subsequently digitized using the microscopic HS system (Fig. 1c), allowing for a detailed analysis of their spectral characteristics. Multiple HS images were acquired to cover the entire selected ROI. Figure 1d shows an example of the annotations within the pathological slide and the selection of different ROIs and the HS images (imaged at 20×). Finally, Fig. 1e summarizes the number of HS images acquired for each patient in the HistologyHSI-GB dataset.

### Microscopic HS system

**Fig. 2:**
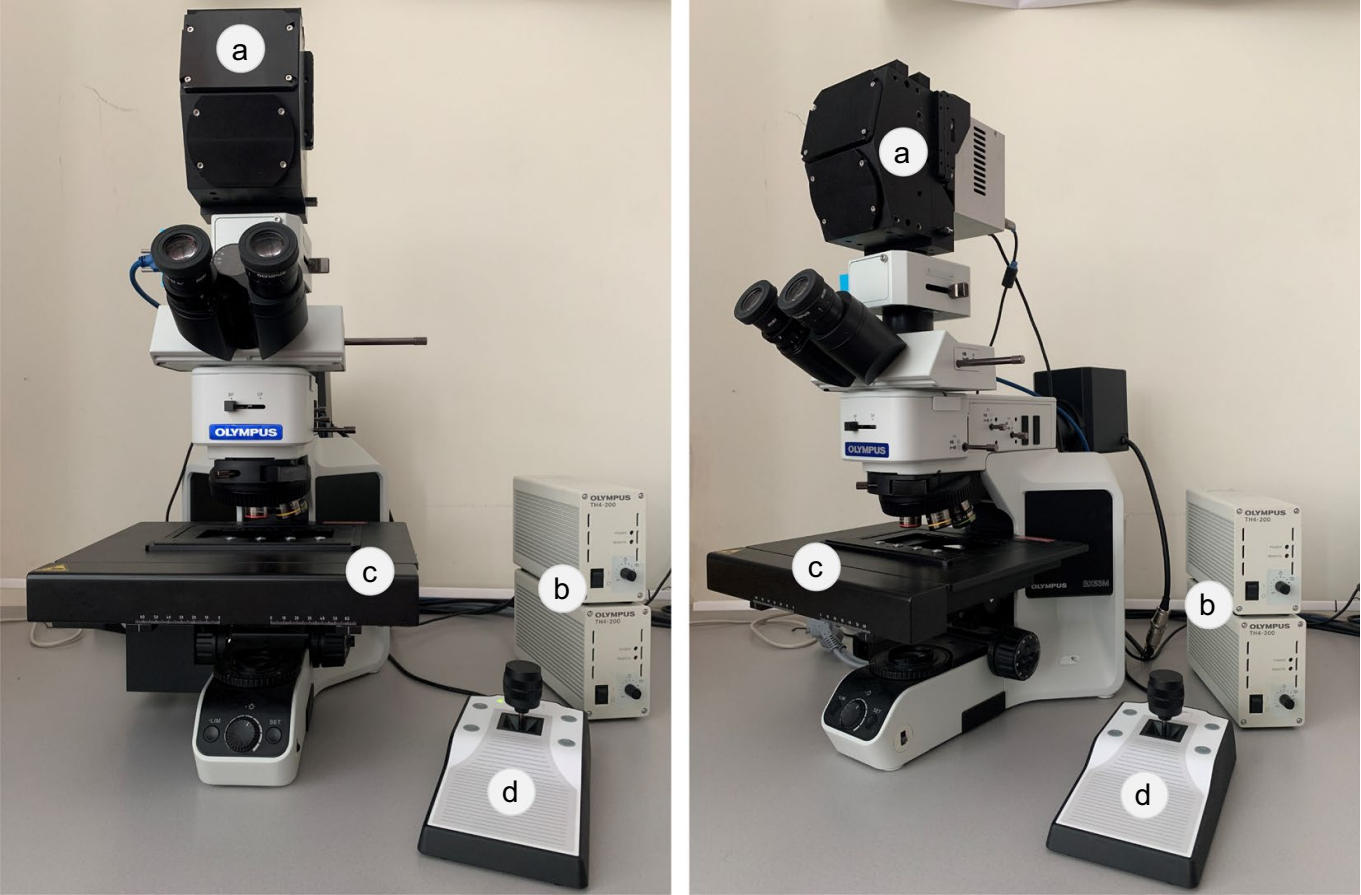
Microscopic HS system. (**a**) HS camera. (**b**) Halogen light source. (**c**) Positioning joystick. (**d**) XY linear stage.

In this study, an HS camera coupled to a conventional brightfield microscope was employed to capture the HistologyHSI-GB dataset (Fig. 2). The HS camera (Fig. 2a) is a Hyperspec^®^ VNIR A-Series from HeadWall Photonics (Fitchburg, MA, USA), which is based on an imaging spectrometer coupled to a CCD (charge-coupled device) sensor, the Adimec-1000 m (Adimec, Eindhoven, Netherlands). This HS camera works in the visual and near-infrared (VNIR) spectral range, from 400 to 1000 nm with a spectral resolution of 2.8 nm, sampling 1004 spatial pixels and, 826 spectral channels. The microscope is an Olympus BX-53 (Olympus, Tokyo, Japan), with four magnification lenses: 5×, 10×, 20× and 50×. The objective lenses are optimized for infrared (IR) observations and the light source is an halogen lamp (Fig. 2b). The HS camera is based on a push-broom technique, requiring a spatial scanning to acquire an HS cube. The system employs a mechanical stage (SCAN, Märzhäuser, Wetzlar, Germany) attached to the microscope for this purpose, which provides accurate movement in the 3 spatial axes directions (Fig. 2c-d). A more detailed description of the different parts of the acquisition system can be found in Table 1. A custom software was developed for synchronizing the scanning movement and the HS camera data acquisition. The optimal exposure time was configured to 40 *ms* (the maximum allowed by the HS camera). The scanning speed of the microscope platform was adjusted according to the ratio of pixel size to exposure time to obtain squared pixels in the resulting HS cubes. Since multiple images were captured from each ROI, the software was designed to enable the acquisition of consecutive HS cubes in a row. Whenever an HS cube was captured (composed by 800 lines), it was stored in memory while the camera and platform continuing to capture data until several cubes were captured. This approach helps save time during image acquisition and minimizes the need for human intervention. To prevent potential degradation of focus or errors caused by the platform while moving, the capture of consecutive HS cubes was limited to a maximum of ten.

**Table 1:** Description of the HS microscopic system components.

	Component	Manufacturer	Model	Key Parameter	
Microscope	Microscope Model	Olympus, Tokyo, Japan	BX-53	Brightfield microscope	
	Trinocular Tube		U-TR30IR-1-2	30° inclination and FN22	
	Eyepiece		WHN10X-H-1-7	Wide field eyepiece FN22	
	Lenses*		LMPLN-IR	5× and 10×	
			LCPLN-IR	20× and 50×	
	Power supply unit		TH4 - 200	12 V 100 W	
	Lamp House		U-LH100IR-1-7	Transmittance and reflectance	
	IR Halogen Lamp	Philips, Amsterdam, Netherlands	7724 EVA	400–1800 nm	
	Stage	Märzhäuser, Wetzlar, Germany	SCAN 130 × 85	3D movement with ± 3 µm resolution	
	Joystick		M-HID-JS-3	Movement in the 3 axes	
	Camera Adapter	Olympus, Tokyo, Japan	U-CMAD3-1-7	C-mount	
HSI System	HS Camera	HeadWall Photonics, Fitchburg, MA, USA	Hyperspec^®^ VNIR A-Series	Technology	Push-broom scanning
				Spectral range	400 to 1000 nm
				N° of bands	826 bands
				Spectral resolution	2.8 nm
				Spatial size	1 × 1004 pixels
	FPA Detector	Adimec, Eindhoven, Netherlands	RA1000m	CCD with 7.4 μm pixel pitch	
	Frame grabber	EPIX, Inc., Buffalo Grove, IL, USA	PIXCI^®^ EL1	PCIe x1 Camera Link Frame Grabber	

### Data acquisition methodology

**Fig. 3:**
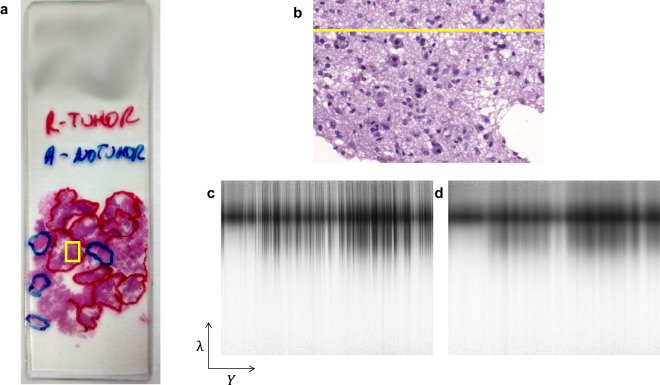
Capture process to obtain focused HS cubes. (**a**) Example of a histology slide with tumour and non-tumour annotations. The yellow square identifies a ROI where the HS image was captured. (**b**) Synthetic RGB image where the *Yλ* frame employed to focus the sample is marked in yellow. Examples of (**c**) focused and (**d**) unfocused *Yλ* frames.

As previously mentioned, relevant areas were identified on the slides and highlighted with a pen in blue (non-tumour) or red (tumour). The capture process of a sample starts by selecting a ROI, from different non-tumour and tumour highlighted areas, to be imaged. Since cells details are needed for further processing, a 20× magnification was chosen to capture the HS images. The coarse focus of the specimen (Fig. 3a) is performed using the microscope binoculars. The procedure relies on the user’s subjective criteria. The final HS image is brought into focus by examining a specific frame captured by the push-broom camera, referred to as the *Yλ* frame (e.g., *Yλ* frame extracted from the yellow line in Fig. 3b). The *λ* axis of an *Yλ* frame corresponds to the spectral information, while the *Y* axis represents the spatial information across the field of view (FOV) of the camera. The objective is to identify the sharpest spatial frequency along various working distances from the sensor to the sample (Fig. 3c shows a focused *Yλ* frame while Fig. 3d shows an unfocused one). The working distance adjustment is performed precisely by using the Z movement with the joystick.

After achieving the optimal focus on the sample, the software was configured to capture several HS images consecutively, where the number of images is defined as an input parameter. The number of images should be kept relatively low to avoid the focus degradation throughout the specimen, due to the non-flat nature of microscopic samples and the platform error/vibration during movement. In this case, a maximum of 10 HS images were extracted consecutively from a selected ROI. The dataset was captured with the light power set to the maximum (100 W) and the exposure time to 40 *ms*. At 20× magnification, the pixel size is 0.373 µ*m*, and the microscope platform was configured to scan the sample at a speed of 9.325 µ*m*/*s*. Furthermore, to overcome the challenges posed by the high dimensionality of the HS images, the collected cubes were constrained to a spatial size of 800 lines, resulting in 1.23 GB data cubes. The HS cubes had a dimension of 800 × 1004 × 826 (number of lines × number of rows × number of bands), corresponding to a spatial size of 299 × 375 µ*m* recorded over a span of 32 *s*.

After the HS images were captured, the reference images for calibration were acquired. In HS image processing, flat-field calibration is an essential step designed to correct the raw data recorded by an HS system. This method corrects the HS data for differences due to the environmental conditions and instrumentation. The flat-field calibration makes use of white (*WR*) and dark (*DR*) reference images. The WR recording is designed to capture data about the HS imaging system under the same conditions used for sample collection, without involving the sample itself. Therefore, the WR is obtained by scanning a section of the histological slide where no tissue is present. Since there is no sample material in such position of the slide, this HS frame contains the maximum values that the sensor is able to measure for each pixel and wavelength in the specified capturing conditions (exposure time, light intensity, the optical properties of the glass slide, etc.). Afterwards, the *DR* is captured by blocking the light transmission to the HS camera. This HS frame contains the minimum values that the system is able to provide for each pixel and band, and also information about the dark currents in the CCD. Ideally, the *DR* values should be very close to zero. However, higher values can be obtained, typically due to the intrinsic noise of the sensor. To ensure a robust measurement of the reference images for calibration, 100 *Yλ* frames are captured for both *WR* and *DR*, allowing any potential errors to be averaged. These reference images were employed for the HS data calibration as detailed in next section. During the acquisition process of the HistologyHSI-GB dataset, image-level annotations were applied. These annotations (tumour or non-tumour) remained consistent across the entire HS cube, indicating that all data within the cube shared the same annotation.

### HS Data calibration

**Fig. 4:**
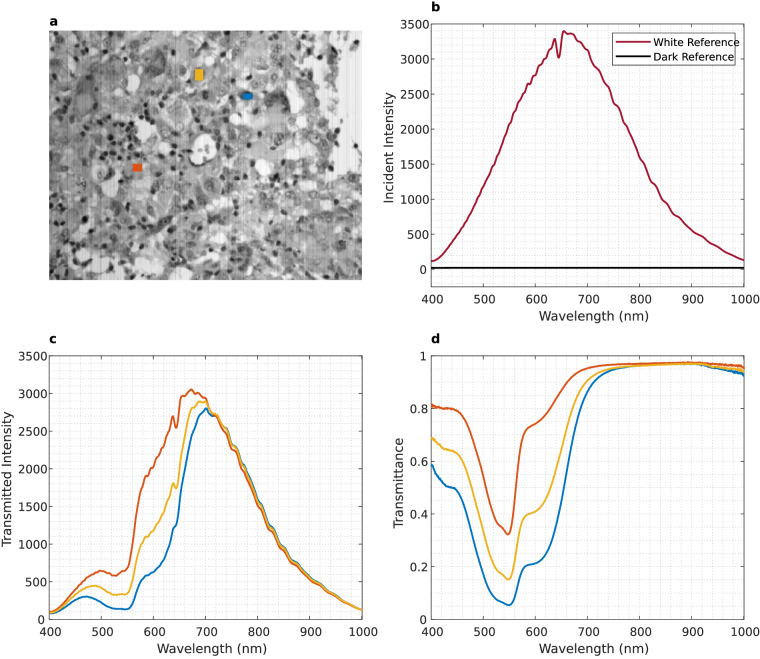
Effect of calibration in the spectral signatures. (**a**) Grayscale image (generated by averaging each spectral band) and selecting pixels corresponding to different materials. (**b**) *WR* and *DR* spectral signatures. (**c**) Uncalibrated spectral signatures from the selected pixels. (**d**) Calibrated spectral signatures from the selected pixels. Colours in (**c,d**) correspond to selected pixels in (**a**).

The goal of the HS microscopic system is to provide a spectral signature per spatial pixel of the captured scene. These spectral signatures indicate the percentage of incident radiation that the scanned object transmits or reflects at each captured wavelength. Various factors, including the inherent spectral response of the sensor, the transmission of light through lenses and optical components, and the spectral characteristics of the light source influence the spectral response of an HS acquisition system. To obtain spectral signatures that accurately indicate the percentage of transmitted or reflected radiation at each wavelength in the sample, the HS cubes need to be calibrated. This calibration consists of normalizing the captured HS pixels by linearly scaling their values considering the *WR* and *DR*. Equation (1) is employed to calibrate the HS data, where *r*_*i*_ and *Raw*_*i*_ refer to each *Yλ* frame from the calibrated and the raw image, respectively. Figure 4 shows an example of how the spectral signatures of different pixels (Fig. 4a) are scaled to transmittance using the aforementioned calibration. The shape of the *WR* and *DR* is shown in Fig. 4b, and several pixels from a ROI before (Fig. 4c), and after calibration (Fig. 4d).

1 $${r}_{i}=\frac{Ra{w}_{i}-DR}{WR-DR}$$

**Fig. 5:**
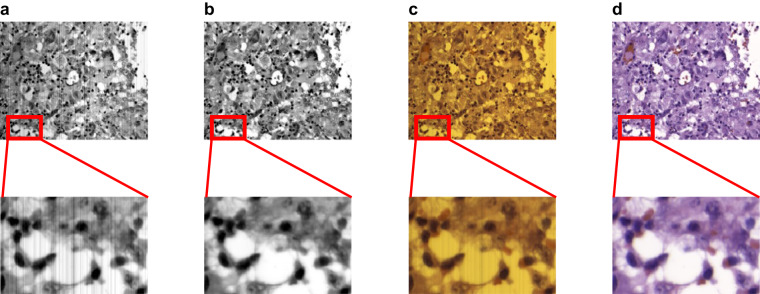
Examples of the uncalibrated and calibrated HS images. (**a,b**) grayscale representation generated by averaging all spectral bands of the uncalibrated and calibrated HS images, respectively. (**c,d**) synthetic RGB image of the uncalibrated and calibrated HS images, respectively, generated using a model of human eye spectral response.

Furthermore, the calibration process also helps to remove the stripping noise effect, which typically appears when acquiring HS images using push-broom scanners^40^. The stripping noise consists in spatially coherent lines that appear in the spatial scanning axis due to static artifacts produced in the sensors, which are repeated in each push-broom frame, as shown in Fig. 5a. In the calibrated images, the effect of the stripping noise disappears (Fig. 5b). The stripping noise is mainly produced due to the fact that different photo-receptors of the sensor have slightly different sensibilities, producing slightly different values when measuring exactly the same amount of incident radiation. The effect of stripping noise and light influence can also be observed in the synthetic RGB shown in Fig. 5c and how this effect disappears after performing the calibration (Fig. 5d).

**Fig. 6:**
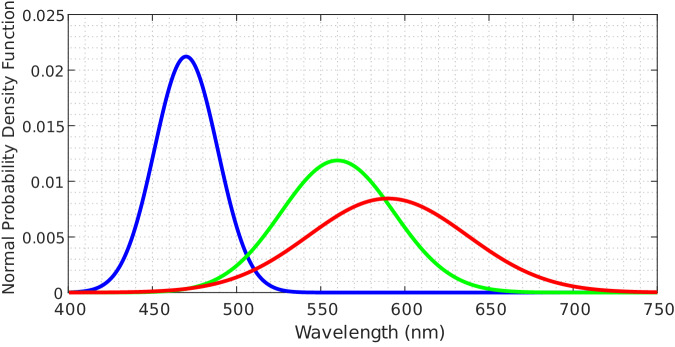
Human eye spectral response to light where different colour line represents the normal probability distribution function modelling each channel.

Greyscale images (Fig. 5a,b) were generated by averaging all spectral bands of the HS image, while the synthetic RGB image (Fig. 5c) was obtained closely mimicking the spectral response of the human eye^41^. For modelling the human eye spectral response, the method employed the normal probability density function following Eq. (2) over the HS data, where *μ* is the mean (*μ*_*R*_ = 590*, μ*_*G*_ = 560, and *μ*_*B*_ = 470) and *σ* is the standard deviation (*σ*_*R*_ = 0.08, *σ*_*G*_ = 0.06, and *σ*_*B*_ = 0.04). In Fig. 6, we can observe that, after the normal probability density function, RGB channels take the following central values and bandwidths: *R* = 590 ± 44 *nm*, G = 560 ± 79 *nm* and, *B* = 470 ± 111 *nm*.

2 $$f(x)=\frac{1}{\sigma \sqrt{2\pi }}{e}^{-\frac{1}{2}{\left(\frac{x-\mu }{\sigma }\right)}^{2}}$$

**Fig. 7:**
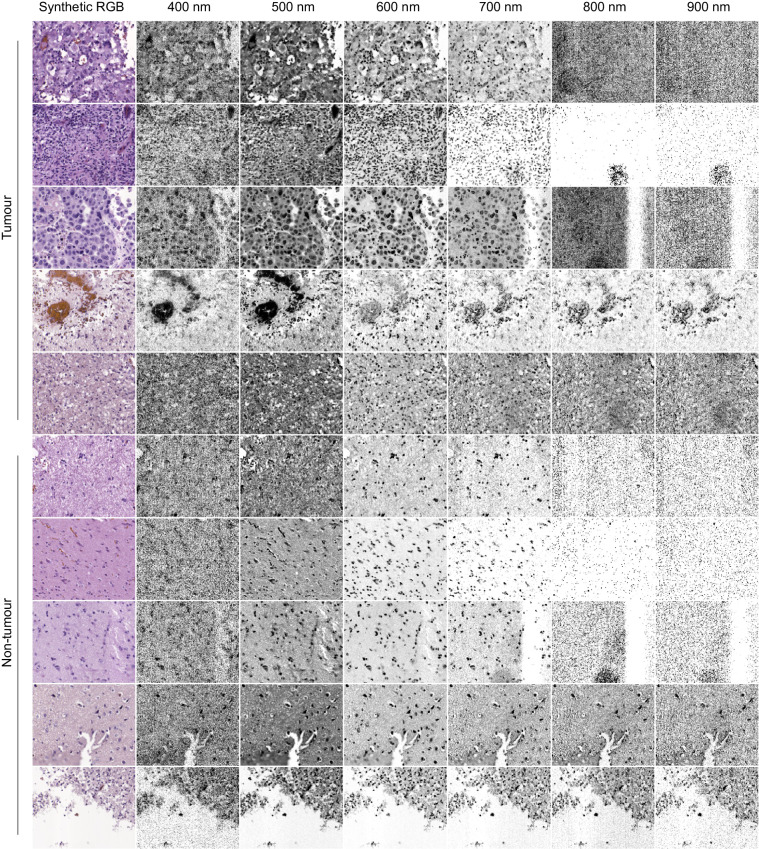
Examples of HS images from the HistologyHSI-GB dataset showing the synthetic RGB images and different spectral bands after calibration for tumour and non-tumour samples.

Finally, in order to present some examples of the HistologyHSI-GB dataset, Fig. 7 shows the synthetic RGB images, as well as the different calibrated spectral bands found in several HS cubes. The contribution of the sensor noise can be observed in the extreme bands.

## Data Records

**Fig. 8:**
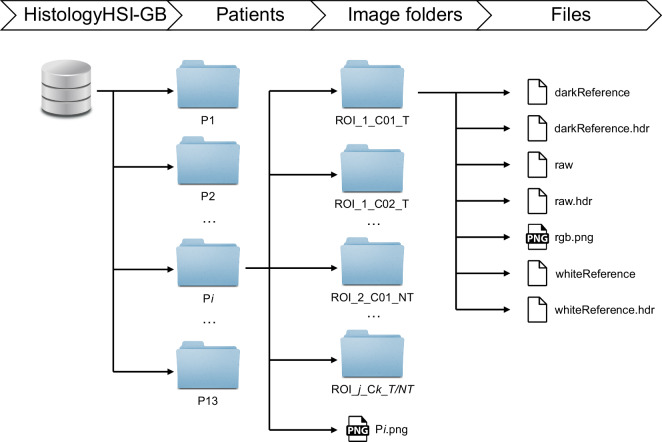
Graphical representation of the HistologyHSI-GB dataset structure.

The HistologyHSI-GB dataset^42^ has been deposited in The Cancer Imaging Archive (TCIA) repository^43^ for cancer imaging. The dataset is structured in a hierarchy of folders, as shown in Fig. 8. At the top level of the hierarchy there is a single folder associated with each one of the patients comprising the dataset. At the patient level, the folder names correspond to *Pi*, where $\{i\in {\mathbb{N}}|1\le i\le 13\}$. For each patient, we can find several folders containing the different HS images for that patient. There is a different number of folders per patient, and the name of each folder encodes the information about which ROI of the histological slide the data was acquired from (*ROI_j*) and another field indicating an image identifier within that ROI (*Ck*). The folders in the image level also contain information about the image-level annotations according to the diagnosis, which can be tumour (*T*) or non-tumour (*NT*). The number of ROIs and image identifiers varies depending on the patient, but the total number of images from each class can be found in Fig. 1e. A conventional image of the slide with the macroscopic annotations and the location of the different ROIs within the slide is available for each patient (*Pi.png*).

Finally, each folder within the image-folder level contains an HS image from the histological slide, the necessary files for the calibration (dark and white references), and a synthetic RGB image extracted from the HS cube. The HS cubes are stored in ENVI format^44^ (the standard format for storing HS images). The ENVI format consists of a flat-binary raster file with an accompanying ASCII (American Standard Code for Information Interchange) header file. A more detailed description of the different files in each image folder can be found Table 2. The HS cubes from the histological slides and the white and dark references are stored as ENVI files. The HistologyHSI-GB dataset comprises 469 images from 13 different patients, where 166 images are labelled as tumour, and 303 are labelled as non-tumour.

**Table 2:** Brief description of the different files contained in each folder in the dataset.

File name	Description
darkReference	ENVI binary file containing the dark reference used for calibration.
darkReference.hdr	ENVI header for the dark reference.
raw	ENVI binary file containing the histological HS data.
raw.hdr	ENVI header for the raw file.
rgb.png	Synthetic RGB image extracted from the HS cube.
whiteReference	ENVI binary file containing the white reference used for calibration.
whiteReference.hdr	ENVI header for the white reference.

## Technical Validation

**Fig. 9:**
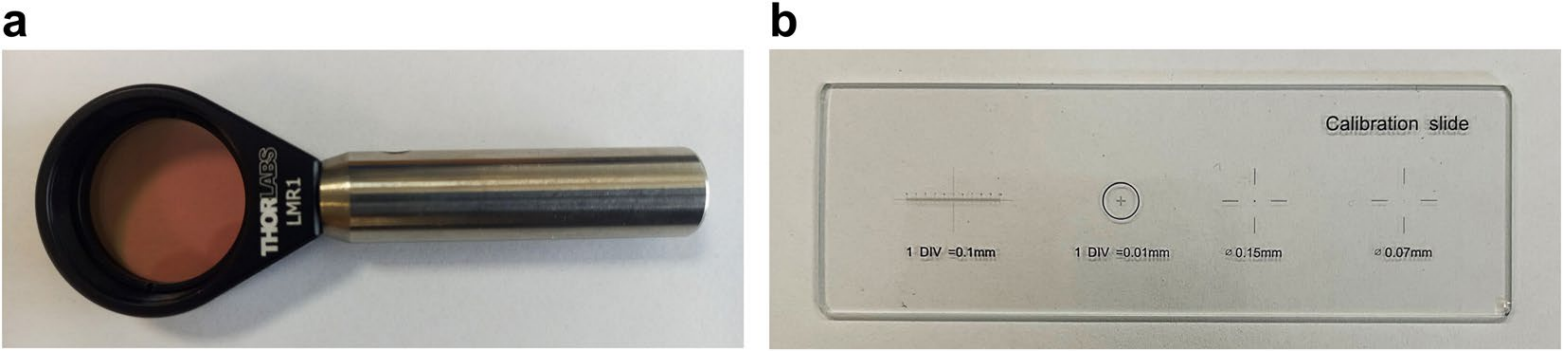
Spectral and spatial calibration targets. (**a**) Certified WCT-2065 polymer. (**b**) 0.01 mm microscope slide reticule.

A technical validation was accomplished to support the quality of the HistologyHSI-GB dataset. Linear sensor systems demonstrate analogous basis functions for both spectral sensitivity and responsivity decomposition^45^. Spectral responsivity refers to the effectiveness of light detection in relation to its frequency or wavelength. However, camera channels often exhibit varying sensitivity across different wavelengths due to the spectral responsivities of the detectors and the non-uniform output of diffractive or filtering elements^46^. Proper characterization is essential for ensuring the reliability and accuracy of HS data analysis and interpretation. HS data captured for noise quantification and spectral and spatial calibration which are used to perform the technical validation (Fig. 9) can be found in a published dataset^47^.

### Signal to noise ratio

**Fig. 10:**
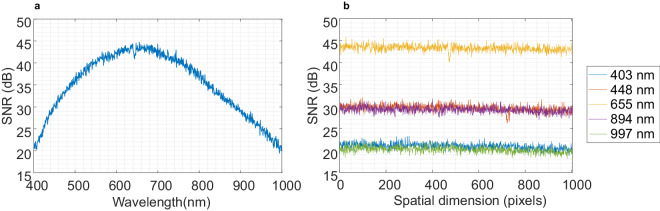
SNR of the microscopic HS system: (**a**) over the spectral range for the central pixel of the push-broom frame and (**b**) its spatial distribution for different wavelengths (blue: 403 nm, orange: 448 nm, yellow: 655 nm, purple: 894 nm and green: 997 nm).

In this section, we present the signal-to-noise ratio (SNR) measurements for our instrumentation. We obtained the signal (S) values by capturing images of the light without any sample, similar to the procedure used for recording the WR in flat-field calibration. For the noise (N) values, we recorded HS images in the absence of light. The SNR was calculated as the ratio between the mean value of S and the standard deviation of N. These recordings were taken over 100 push-broom frames under the same conditions as the image recordings. We calculated the SNR over the entire spectral range for the central pixel of the push-broom frame (Fig. 10a), which shows that the SNR exceeds 20 dB over the entire spectral range, peaking 42 dB at 655 nm. Furthermore, the SNR remains above 30 dB for wavelengths ranging from 448 to 894 nm. The SNR spatial distribution was also calculated over the camera FOV for different spectral bands (Fig. 10b), showing that SNR is evenly distributed over the FOV for the different spectral bands, indicating a uniform spatial distribution.

### Spectral characterization

The WCT-2065 polymer (Fig. 9a), a transmittance wavelength calibration standard from Avian Technologies (New London, USA), was employed to conduct the spectral characterization of the microscopic HS system. It represents an alternative designation for NIST (National Institute of Standards and Technology) SRM-2065 standard^48^. Its purpose lies in facilitating the calibration of spectrophotometers, covering the wavelength range of 400–2200 nm. The standard uses a glass filter material that incorporates a combination of rare earth oxides. This glass composition includes holmium oxide, samarium oxide, ytterbium oxide, and neodymium oxide, which are blended with lanthanum, boron, silicon, and zirconium oxides found in the base glass. The resulting combination of these oxides creates a filter material with specific optical properties suitable for calibration purposes.

**Fig. 11:**
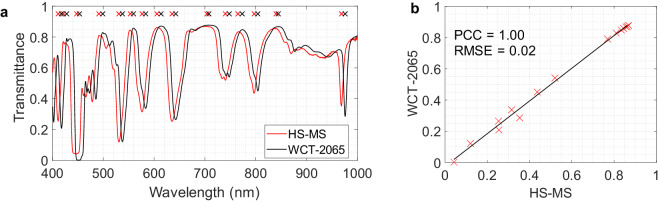
Spectral characterization of the microscopic HS system. (**a**) Manufactured certified spectral signature of the WCT-2065 polymer (black line) and spectral signature captured by the microscopic HS system (red line). (**b**) Pearson Correlation Coefficient between WCT-2065 and the measured HS peak values.

An HS image of the WCT-2065 polymer was captured using the microscopic HS system and further pre-processed. This calibration standard can qualitatively validate the spectral quality of the employed instrumentation (Fig. 11). However, a systematic approach is required for a more accurate and thorough calibration. In order to perform the quantitative validation, the Pearson correlation coefficient (PCC) and root mean square error (RMSE) Eq. (3) were employed to measure the difference between two sets of data. PCC measure the degree of linear anti-correlation or correlation in the range [−1, 1], where −1 indicates perfectly linearly anti-correlated data and 1 indicates perfectly linearly correlated data and it is computed following the Eq. (4). In addition, local maxima and minima were found to detect the most significant signal peaks. Thus, similar peaks were identified both in the captured image and the reference (represented by red and black crosses in Fig. 11a, respectively), resulting in a mean wavelength difference spectra shift of 6.60 *nm* between them. Furthermore, Fig. 11b shows that the PCC between WCT-2065 and the measured HS peak absorbance values provided good value, as well as, it RMSE (*PCC* = 1 and *RMSE* = 0.02). Thus, this NIST traceable standard allows accurate and reliable measurements of the spectral reliability of the HS acquisition system.

3 $$RMSE=\sqrt{\frac{{\sum }_{i=1}^{N}{({y}_{i}-{\hat{y}}_{i})}^{2}}{N}}$$

4 $$PCC=\frac{\sum ({x}_{i}-\bar{x})({y}_{i}-\bar{y})}{\sqrt{{\sum ({x}_{i}-\bar{x})}^{2}{\sum ({y}_{i}-\bar{y})}^{2}}}$$

### Spatial characterization

Spatial resolution, the ability of a camera to capture fine details and distinguish between separate objects, is also a critical feature in imaging systems. It determines the smallest size of an object that can be recorded. This parameter is essential in applications like histological diagnosis, where identifying small details is essential. Accurate spatial resolution characterization enables improved system performance and precise analysis in various fields, including histopathology^49^. Firstly, the camera is manually aligned to capture the information properly^50^. Then, the spatial resolution of the microscopic HS system was evaluated both theoretically and empirically. The theoretical calculation of the FOV, shown in Eq. (5), considered factors such as pixel size (*Ps*), number of pixels (*N*), magnification (*M*_*i*_), and sensor size (*S*_*s*_).

5 $$FOV=\frac{Ps\cdot N}{{M}_{i}}=\frac{{S}_{s}}{{M}_{i}}$$

**Fig. 12:**
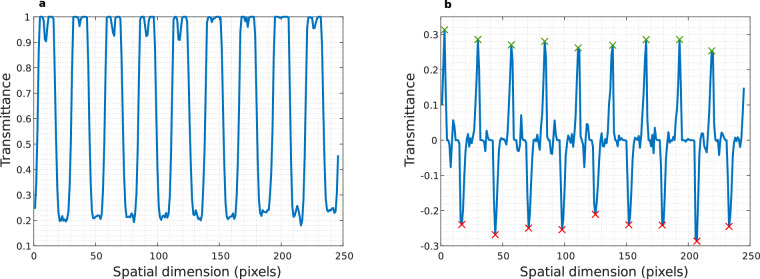
Pixel size validation using a micrometre ruler. (**a**) Profile of *Yλ* frame extracted from the *Yλ* frame and (**b**) its first derivative where red crosses are local minima peaks and green crosses local maxima peaks.

An empirical test using a micrometre ruler (Fig. 9b) provides further insight into the spatial resolution capabilities of the cameras. In order to perform this test, a *Yλ* spatial profile of the ruler (Fig. 12a) and its first derivative (Fig. 12b) was analysed to determine the mean distance between peaks (local minima peaks signalled with red crosses and local maxima peaks with green crosses). The results of the theoretical calculation provide a pixel size of 0.3700 *μm* and the empirical one is 0.3697 *μm*. This method confirmed that the spatial resolution of the microscopic HS system matches the theoretical pixel size with an average error of less than 0.0003 *μm*.

Once the pixel size has been calculated and the camera has been visually aligned, together with the information about the mechanical resolution and frame rate of the camera, the required motor rotation speed of the mechanical stage is determined. However, an additional analysis was conducted to further improve and verify the correct configuration of the scanning parameters. The entire HS acquisition system is considered as a whole, including the microscope, camera, and movement mechanism. For this evaluation, the goal is to capture an image of a circle printed in a calibration slide (*dot target*) and evaluate its appearance to identify camera misalignments and suboptimal movement speeds. The circle appears as a perfect rounded circle when captured at the correct speed but appears as an ellipse when the speed is too high or too low. While visual inspection provides a relatively good assessment, an automatic methodology^50^ is needed for a more precise and rigorous calibration. First, principal component analysis (PCA) is employed to find the directions of the longest and shortest axes of the ellipse (*ϕ*_*min*_ and *ϕ*_*max*_). Then, eccentricity can be calculated following Eq. (6), where a perfect circle would provide values close to zero. In our case, the dot target from the calibration slide was captured, and its eccentricity was computed providing accurate results (*e* = 0.176). Thus, the microscopic HS system is properly calibrated in the spatial domain, and it is possible to acquire HS images under satisfactory conditions.

6 $$e=\sqrt{1-\frac{{({\phi }_{min})}^{2}}{{({\phi }_{max})}^{2}}}$$

## Usage Notes

### Recommended pre-processing

The pre-processing framework applied to each HS cube is based on standard calibration and spectral band reduction. First, HS images are transformed from radiance to normalized transmittance by calibration. As a result of the strong correlation of spectral information between adjacent spectral bands, we propose to reduce the spectral dimensionality of the original data. A spectrally reduced HS image is generated by averaging the spectral bands of adjacent neighbouring bands to perform this band reduction. Using a spectral window of three neighbours, this process reduces the original 826 bands to 275, while slightly decreasing the presence of white Gaussian noise. Furthermore, reducing the number of bands proves to be advantageous in terms of reducing the computational cost of subsequent image processing tasks. However, this band reduction is optional, depending on the further processing interest. Additionally, for image analysis involving the spectral analysis of the samples, it is recommended to perform a background sample segmentation, where the pixels corresponding to the tissue and the background light of the microscope are identified. Finally, to use classification methods, the label (*tumour* or non-tumour) of each HS cube should be extracted from the folder name.

### Recommended data partition and data HS processing applications

To perform machine learning analysis, an unbiased data partition should be performed. The dataset used for this study poses three challenges. First, the dataset is limited in the number of patients (13 patients). Second, samples containing both classes (tumour and *non*-tumour) are only available for 8 patients. Hence, the non-tumour samples information is limited in terms of patients. Third, the dataset is unbalanced, with more images annotated as non-tumour. In previous works^36,38^, a data partition based on 4 different folds was employed. Furthermore, spectral unmixing techniques could be performed as a preprocessing stage prior to classification^51^, or they can be used to determine the abundances of known endmembers of the images, specifically identifying the proportions of the H&E stains in each pixel^52^.

### Limitations and future perspectives

The dataset has several limitations. As previously mentioned, the primary limitation is its relatively small cohort, consisting of data from only 13 patients. Furthermore, information for both classes of interest, tumour, and non-tumour, is available for only 8 of these patients. This leads to an imbalanced dataset, with a predominance of images classified as non-tumour. Such an imbalance could potentially introduce bias and affect the generalizability of the findings derived from this dataset.

Another limitation is related to the type of annotations available in this dataset. The macroscopic annotations of tumour and non-tumour regions on the pathological slides, leading to only image-level annotations for the HS images. A more sophisticated method for digitally annotating the images would allow to identify regions where tumour and non-tumour tissues are adjacent, making possible to capture regions comprising both classes in a single HS image. More detailed digital annotation would help in further validating the classification algorithms on a pixel-by-pixel basis and could also offer potential for other methods such as unsupervised learning or spectral unmixing. However, more detailed annotations would significantly increase the time and manual effort required to label each image.

Finally, this dataset is focused on images captured on a single magnification (20×). The motivation of using the higher magnification available for the instrumentation was driven by the need to capture detailed cell-level information from the histological slides. However, creating a dataset containing the same images at different magnifications could be of potential interest and benefit to the scientific community.

In summary, future datasets of HS histological samples will need to include a larger number of patients, ensure a balanced representation of the various classes of interest, incorporate more detailed annotations, and provide images at various magnification levels.

## Data Availability

A tutorial on how to read and display HS data is available in a public repository: https://github.com/HIRIS-Lab/HistologyHSI-GB. These tutorials include the use of custom MATLAB and Python functions and some of the most common toolbox/libraries.
