# Publisher Correction: Histological Hyperspectral Glioblastoma Dataset (HistologyHSI-GB)

**DOI:** 10.1038/s41597-024-03654-w

**Published:** 2024-07-29

**Authors:** Samuel Ortega, Laura Quintana-Quintana, Raquel Leon, Himar Fabelo, María de la Luz Plaza, Rafael Camacho, Gustavo M. Callico

**Affiliations:** 1 Seafood Industry Department, Norwegian Institute of Food, Fisheries and Aquaculture Research (Nofima), Tromsø, Norway; 2 Institute for Applied Microelectronics, University of Las Palmas de Gran Canaria, Las Palmas de Gran Canaria, Spain; 3 Department of Mathematics and Statistics, UiT The Arctic University of Norway, Tromsø, Norway; 4 Fundación Canaria Instituto de Investigación Sanitaria de Canarias (FIISC), Las Palmas de Gran Canaria, Spain; 5 Research Unit, Hospital Universitario de Gran Canaria Doctor Negrín, Las Palmas de Gran Canaria, Spain; 6 Department of Pathological Anatomy, Hospital Universitario de Gran Canaria Doctor Negrín, Las Palmas de Gran Canaria, Spain

**Keywords:** Optical spectroscopy, Image processing

Correction to: *Scientific Data* 10.1038/s41597-024-03510-x, published online 24 June 2024

In this article the graphics relating to Figs 10 and 11 were inadvertently swapped. The original article has been corrected.

